# Nanotechnology in respiratory medicine

**DOI:** 10.1186/s12931-015-0223-5

**Published:** 2015-05-29

**Authors:** Albert Joachim Omlor, Juliane Nguyen, Robert Bals, Quoc Thai Dinh

**Affiliations:** Department of Experimental Pneumology and Allergology, Saarland University Hospital and Saarland University Faculty of Medicine, Kirrberger Strasse, Geb. 61.4, 66421 Homburg/Saar, Germany; Department of Pharmaceutical Sciences, School of Pharmacy and Pharmaceutical Sciences, SUNY Buffalo, New York, USA; Department of Internal Medicine V, Pneumology, Allergology and Respiratory Critical Care Medicine, Saarland University Faculty of Medicine, Homburg/Saar, Germany

**Keywords:** Nanoparticles, Lung, Airways, Nanotoxicology, Biodistribution, Nanomedicine

## Abstract

Like two sides of the same coin, nanotechnology can be both boon and bane for respiratory medicine. Nanomaterials open new ways in diagnostics and treatment of lung diseases. Nanoparticle based drug delivery systems can help against diseases such as lung cancer, tuberculosis, and pulmonary fibrosis. Moreover, nanoparticles can be loaded with DNA and act as vectors for gene therapy in diseases like cystic fibrosis. Even lung diagnostics with computer tomography (CT) or magnetic resonance imaging (MRI) profits from new nanoparticle based contrast agents. However, the risks of nanotechnology also have to be taken into consideration as engineered nanomaterials resemble natural fine dusts and fibers, which are known to be harmful for the respiratory system in many cases. Recent studies have shown that nanoparticles in the respiratory tract can influence the immune system, can create oxidative stress and even cause genotoxicity. Another important aspect to assess the safety of nanotechnology based products is the absorption of nanoparticles. It was demonstrated that the amount of pulmonary nanoparticle uptake not only depends on physical and chemical nanoparticle characteristics but also on the health status of the organism. The huge diversity in nanotechnology could revolutionize medicine but makes safety assessment a challenging task.

## Introduction

**Fig. 1:**
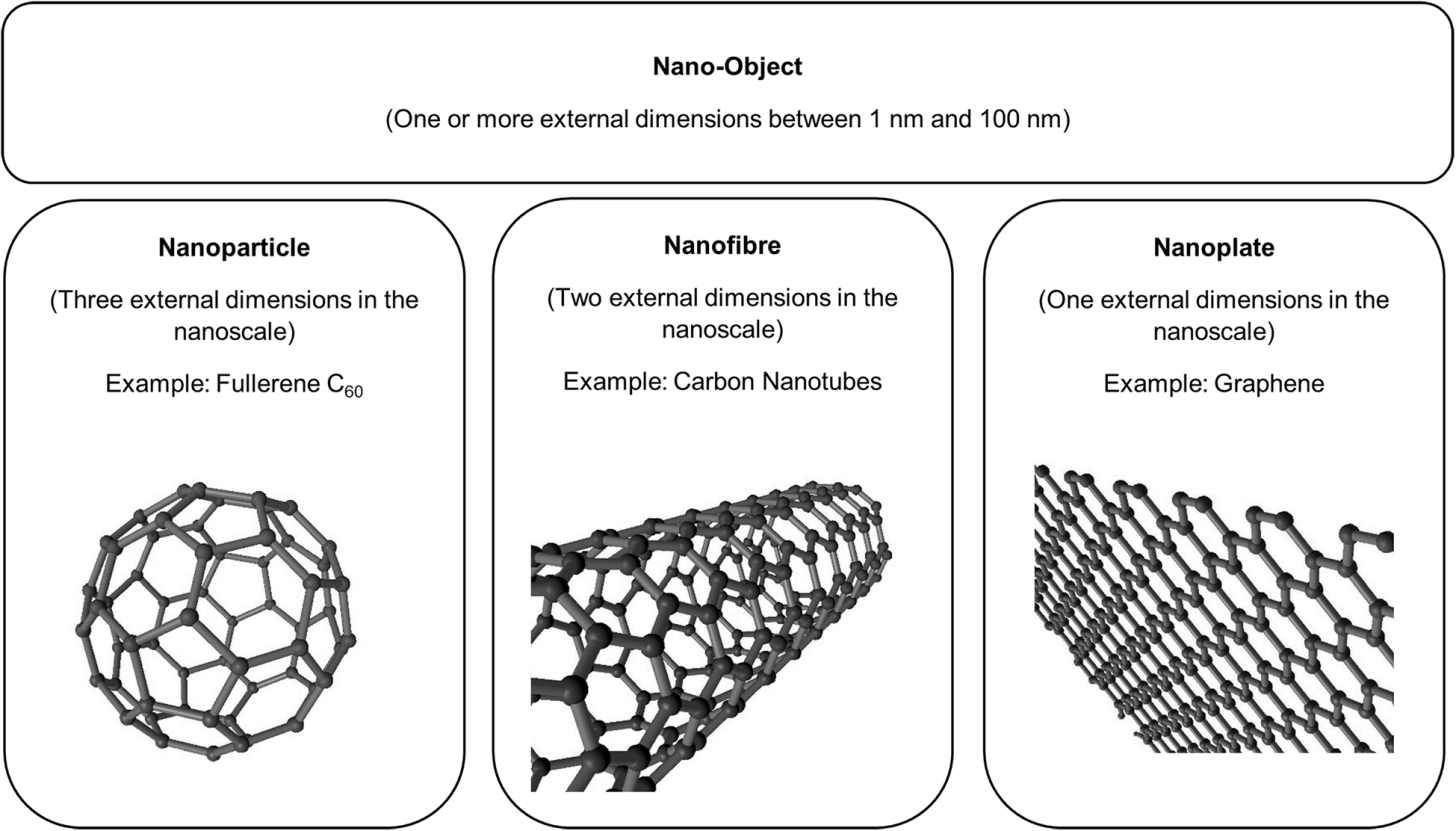
Nano-Objects can be divided into nanoparticles, nanofibres and nanoplates depending on the number of external dimensions in the nanoscale

**Fig. 2:**
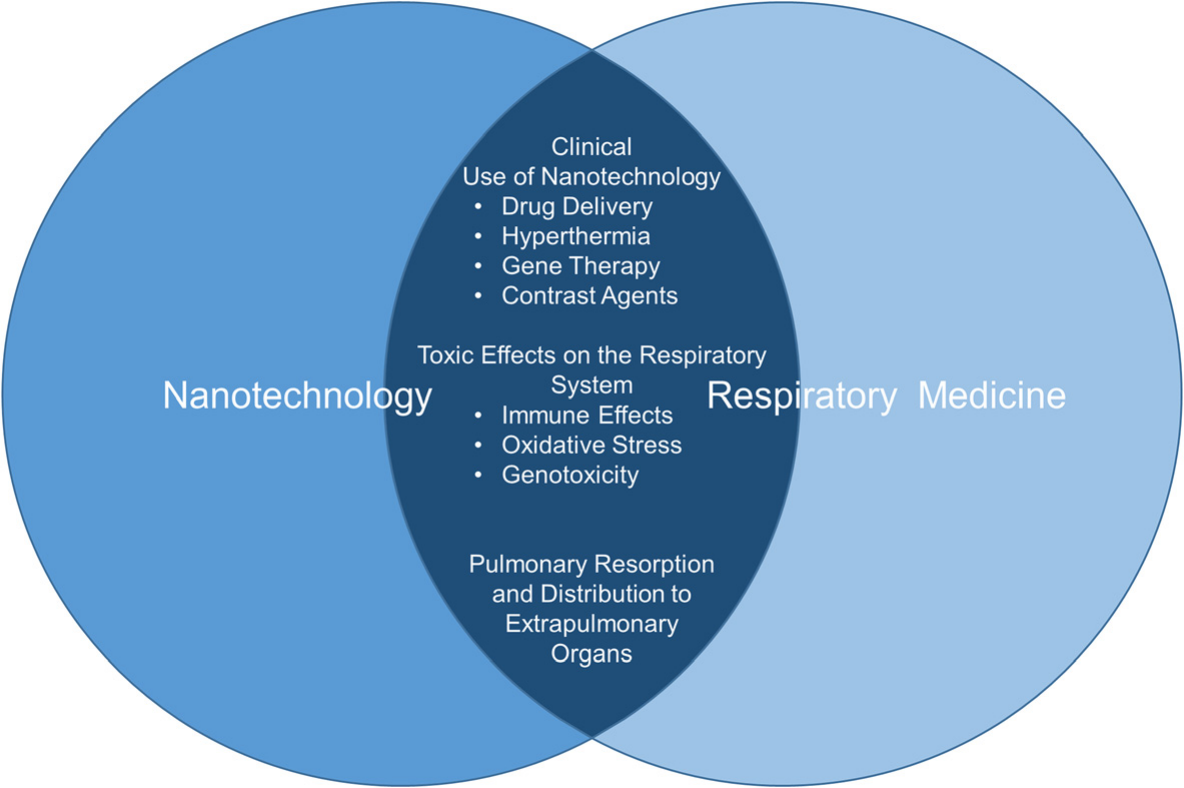
The increasing use of nanotechnology affects respiratory medicine in three main areas. Firstly, nanotechnology enables more sophisticated options in therapy and diagnostics. Secondly, the use of nanomaterials can cause toxic effects in the respiratory system. Health risks associated with the use of nanomaterials are not fully understood and merit further investigation. Moreover, it will be essential to understand the effects of inhaled nanoparticles on extrapulmonary organs

Over the past years nanomaterials have found their way into more and more areas of life. Examples are new coatings and pigments, electronic devices as well as cosmetic products like sunscreens and toothpastes. On top of that, much effort is done to adopt nanotechnology for the treatment of human diseases. The term “Nano” refers to structures in the range of 1 to 100 nm. In contrast to nanoparticles, which have to measure between 1 and 100 nm in all dimensions, nanomaterials may consist of elements bigger than 100 nm but need to be structured in the nanoscale and exhibit characteristic features associated with their nanostructure [1]. In this context, the International Organization for Standardization defined the term nano-object as a material with one, two or three external dimensions in the nanoscale [2] (Fig. 1). Nanomaterials have an extremely high surface area to volume ratio. Therefore, some of them are very reactive or catalytically active. Moreover, in the nanoworld quantum effects become visible and lead to some of the unique properties of nanoparticles. Like viruses and cellular structures, some nanoparticles are able to self-assemble to more complex structures [3]. This makes them interesting candidates for novel drugs. On the other hand it is necessary to redefine toxicology because of nanotechnology. Unlike classical toxicology, where dose and composition matter, in nanotoxicology the focus has to be set on properties like morphology, size, size distribution, surface charge, and agglomeration state as well. Nanotechnology is important for respiratory medicine for several reasons. Firstly, it offers new approaches to treat diseases of the respiratory tract. However, as nanotechnology usage in consumer products, cosmetics, and medicine is continuously increasing, it is also pivotal to understand potentially adverse effects of nanomaterials on the respiratory system. Additionally, studying respiratory effects of manufactured nanomaterials helps to understand the impact of combustion exhaust and ultra-fine dusts on human health. On top of that, the lung is probably the most important gateway of nanoparticles to the human organism. For the assessment of safety in nanotechnology it is therefore also important to elucidate which nanoparticle properties determine pulmonary resorption and biodistribution (Fig. 2).

### Applications of nanotechnology in therapeutics and diagnostics

Although clinical application of nanotechnology in therapeutics and diagnostics is still rare, there are multiple promising candidates for future use in the field of respiratory medicine.

#### Drug delivery

**Fig. 3:**
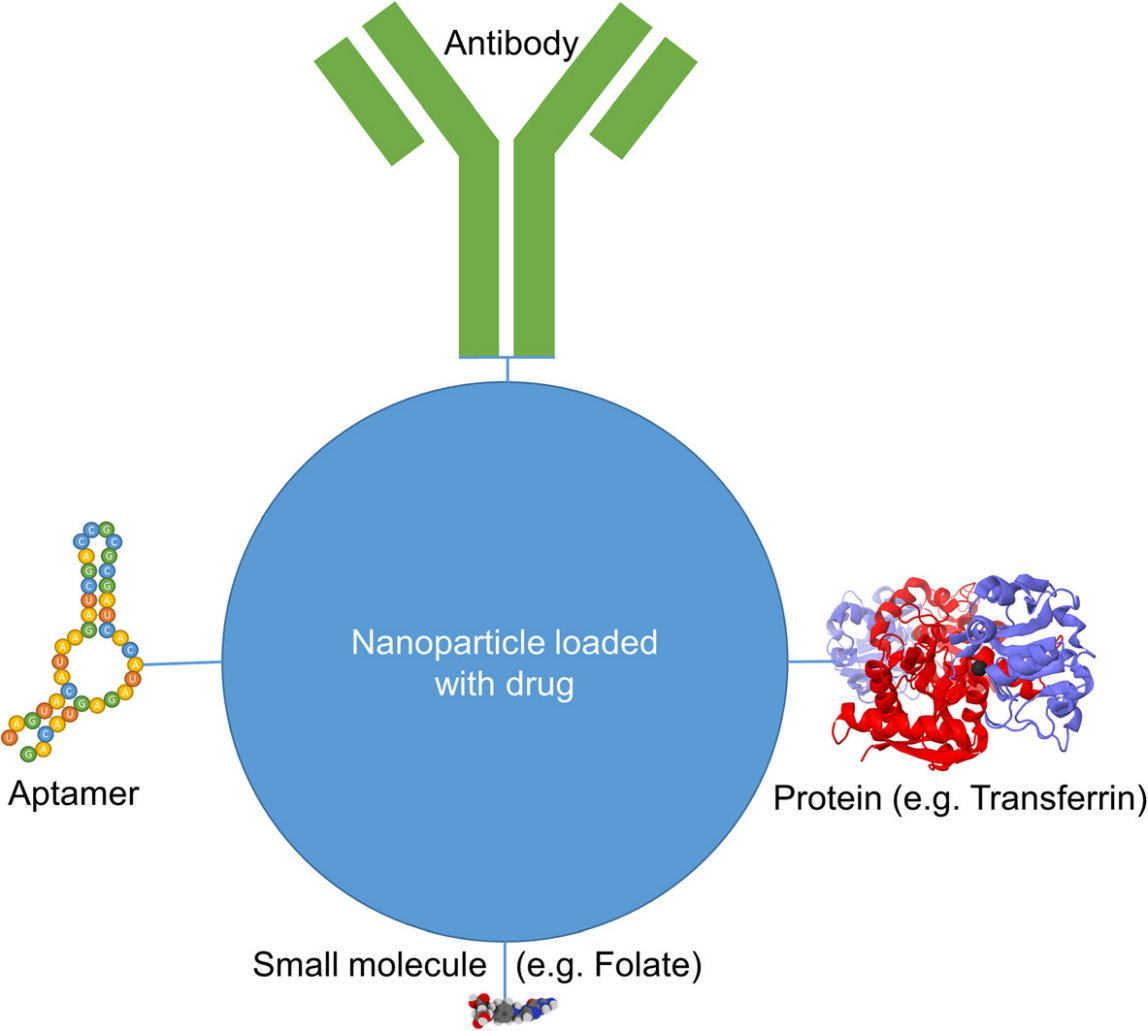
Four different strategies for active targeting of nanoparticle based drug delivery systems are shown. The nanoparticles can be conjugated with tumor specific antibodies or aptamers. Additionally, small molecules, such as folate, as well as proteins, such as transferrin, can be used for targeting receptors that are overexpressed on tumors

Nanoparticles can act as vessels for drugs because they are small enough to reach almost any region of the human organism. Drugs can be bound chemically to the nanoparticles by a multitude of different linker molecules or by encapsulation. This allows better control of toxicokinetics. However, the main advantage is the capability of targeted drug delivery. The targeting can be active or passive. In case of tumor diseases, the leaky and immature vasculature of fast growing tumors can be taken advantage of in order to achieve passive targeting of chemotherapeutic loaded nanoparticles. This is called the enhanced permeability and retention (EPR) effect [4]. The first generation nano drug delivery systems rely entirely on the EPR effect. One example is Genoxol-PM, a polymeric paclitaxel loaded poly(lactic acid)-block-poly(ethylene glycol) micelle-formulation [5]. This nanocarrier has recently been tested in a phase II trial in patients with advanced non-small cell lung cancer (NSCLC). 43 patients were treated with four 3-week cycles of Genexol-PM at 230 mg/m^2^ on day 1 combined with gemcitabine 1000 mg/m^2^ on day 1 and day 8. With a response rate of 46.5 %, the therapy showed favorable antitumor activity. Moreover, emetogenicity was low. However, frequent grade 3/4 adverse events like neutropenia and pneumonia were observed [6]. The second generation nanoparticle drug delivery systems possess targeting ligands. These can be antibodies, aptamers, small molecules and proteins (Fig. 3). The attached ligands actively guide the nanoparticles and therefore the drugs to the tumor cells. Tumor specific monoclonal antibodies are already widely used in cancer therapy. Those antibodies can be attached to nanoparticles for active targeting. In a recent study polyglycolic acid nanoparticles, that were conjugated with cetuximab antibodies for targeting and loaded with the drug paclitaxel palmitate, were administered intravenously to mice with A549-luc-C8 lung tumors. The survival rate of these mice increased significantly compared to the control group [7]. Another approach involves aptamers as targeting agents. Aptamers are synthetic oligonucleotides that are capable of binding specific target structures. Their small size, their simple synthesis, and their lack of immunogenicity make them promising ligands for nanoparticles. Moreover, small molecules such as folate can be used for targeting tumor cells that express a high density of folate receptors. In addition, tumors often overexpress receptors for several proteins. Proteins like transferrin therefore are common targeting ligands [8]. These second generation nanocarriers are already used clinically against lung cancer with substances like Aurimmune Cyt-6091 and Bind-014. Aurimmune Cyt-6091 is a drug delivery system based on gold nanoparticles functionalized with polyethylene glycol (PEG) and tumor necrosis factor alpha (TNF-α). It has been used against adenocarcinoma of the lung in a phase I clinical trial. The TNF-α serves both as targeting and therapeutic agent in this case [9]. A phase II clinical trial for non-small cell lung cancer patients has been planned [10]. The nano drug delivery system Bind-014 is currently tested in a phase II clinical trial as second-line therapy for patients with non-small cell lung cancer [11]. Bind-014 nanoparticles consist of a polylactic acid (PLA) core, in which the anti-tumor drug docetaxel is physically entrapped. The particles are surface-decorated with PEG to reduce elimination from the immune system and contain ligands against prostate-specific membrane antigen (PSMA) for targeting. PSMA is expressed in prostate cancer cells and in the neovasculature of nonprostate solid tumors, such as NSCLC [12]. Preliminary data demonstrates, that Bind-014 is clinically active and well tolerated. It also showed promising effects on patients with KRAS mutations, where ordinary anti-tumor agents usually fail. Additionally, adverse effects like anemia, neutropenia and neuropathy were significantly reduced compared to solvent based docetaxel [13].

Nanoparticle based drug delivery also offers potential in other fields of respiratory medicine. In experiments with tuberculosis infected guinea pigs, it was demonstrated that inhaled alginate nanoparticles encapsulating isoniazid, rifampicin, and pyrazinamide showed better bioavailability and higher efficiency than oral drug medication [14]. Similar results were presented by Pandey et al. with the three antitubercular drugs encapsulated in poly (DL-lactide-co-glycolide) nanoparticles [15]. Moreover, another study demonstrated that pirfenidone loaded nanoparticles have higher anti-fibrotic efficacy in the treatment of mice with bleomycin-induced pulmonary fibrosis than dissolved pirfenidone [16].

#### Hyperthermia

**Fig. 4:**
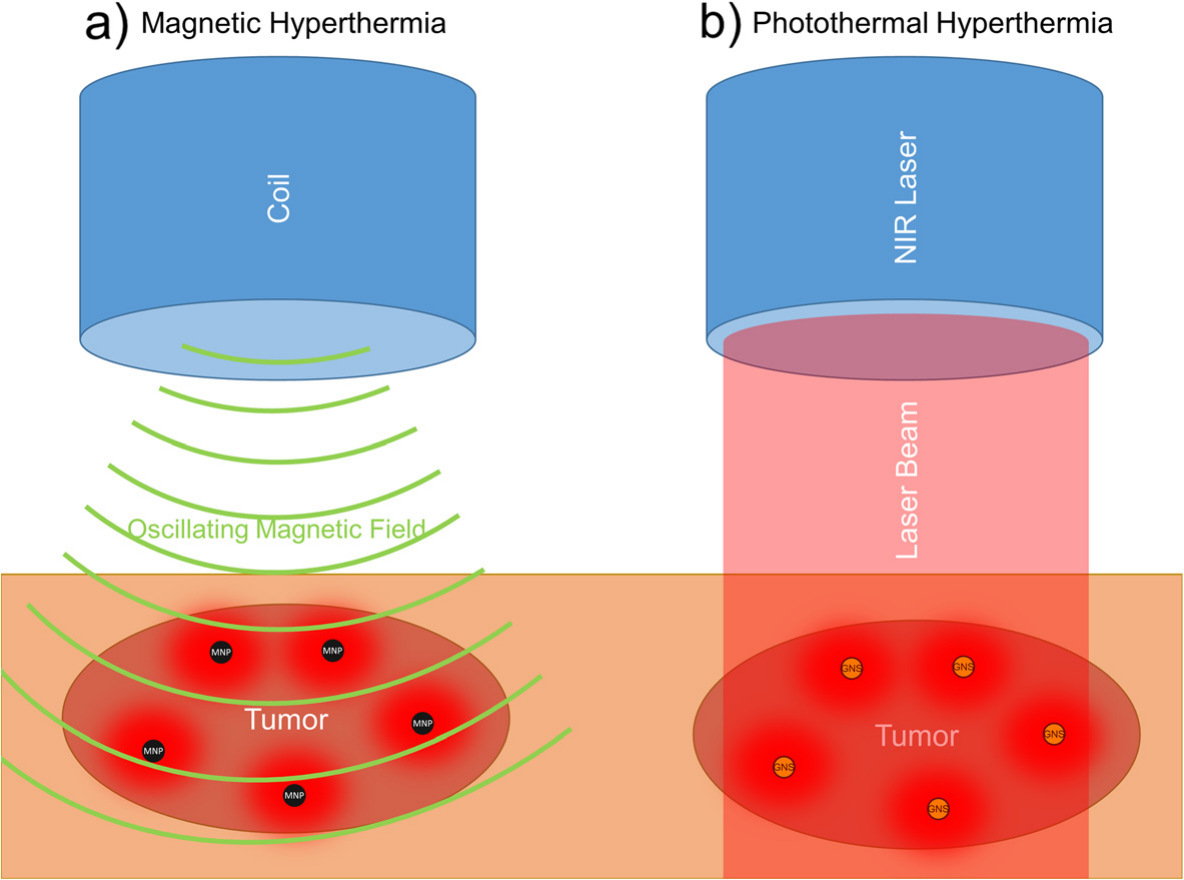
Two different approaches of nanoparticle based hyperthermia therapy are shown. **a** In magnetic hyperthermia, magnetic nanoparticles (MNP) are applied intravenously and accumulate inside the tumor. When an oscillating magnetic field is created by an extracorporeal coil the magnetic nanoparticles produce heat inside the tumor. **b** In photothermal hyperthermia, gold nanoshells (GNS) or similar photosensitizing nanoparticles are applied intravenously and accumulate inside the tumor. Upon exposure of the tumor to near infrared (NIR) laser radiation, the gold nanoshells convert the laser light into heat

Nanoparticle induced hyperthermia can be used to locally destroy tumor cells. Heat generation is usually achieved by two approaches, magnetic and photothermal hyperthermia. In magnetic hyperthermia, an extracorporeal coil creates an alternating magnetic field that heats magnetic nanoparticles inside a tumor. This increases the temperature in the tumor without affecting healthy tissue. A recent study assessed the effect of inhalable superparamagnetic iron oxide nanoparticles in a mouse model of NSCLC. Compared to the non-targeted nanoparticles, the epidermal growth factor receptor (EGFR) targeted nanoparticles showed significantly more effective tumor shrinkage after magnetic hyperthermia treatment [17]. The other approach, photothermal therapy uses laser radiation in the visible or near infrared spectrum and photosensitizing nanoparticles such as gold or graphene. A commercial product called auroshell is available for tumor therapy. Auroshell nanoparticles consist of a silica core surrounded by a thin layer of gold. The gold nanoshells are administered intravenously and accumulate in the tumor due to the EPR effect. Upon exposure of the tumor to a near infrared laser, the laser energy is efficiently converted to heat by the gold nanoshells [18]. This therapy, which is called AuroLase, is currently undergoing clinical trial in patients with primary and/or metastatic lung tumors [19] (Fig. 4).

#### Gene therapy

Like viruses, nanoparticles can be used as vectors for genes. But in contrast to viruses, they are less immunogenic and have higher DNA transport capacity. In a study, DNA loaded polyethylenimine nanoparticles were used in order to treat lipopolysaccharide induced acute lung injury in mice. After intravenous injection of the nanoparticles, the beta2-Adrenic Receptor genes in the nanoparticles led to a short lived transgene expression in alveolar epithelia cells. As a result the 5-day survival rate improved from 28 % to 64 %. The severity of the symptoms measured by alveolar fluid clearance, lung water content, histopathology, bronchioalveolar lavage cellularity, protein concentration, and inflammatory cytokines was also significantly attenuated [20]. DNA loaded nanoparticles are also promising candidates in the treatment of cystic fibrosis. It was shown in a clinical trial that nasal application of DNA nanoparticles is safe and evidently leads to vector gene transfer [21]. One major problem in this context is to overcome the mucus barrier. In a recent study, it was demonstrated that densely PEG-coated DNA nanoparticles can rapidly penetrate extracorporeal human cystic fibrosis and extracorporeal mouse airway mucus. In addition, those particles exhibited better gene transfer after intranasal administration to mice than conventional carriers [22].

#### Diagnostics

Nanoparticles have the potential to improve pulmonary x-ray diagnostics. Folic acid-modified dendrimer-entrapped gold nanoparticles were utilized as imaging probes for targeted CT imaging. In in-vitro and in-vivo tests, the nanoparticles were trapped in the lysosomes of folic acid receptor expressing lung adenocarcinoma cells (SPC-A1). It was possible to detect the tumor cells by micro-CT imaging after nanoparticle uptake. In addition, it was also shown that the particles possess good biocompatibility, with no impact on cell morphology, viability, cell cycle, and apoptosis [23]. Nanoparticles can also be used to enhance MR diagnostics of lung tissue. In experiments with intratracheal administration of Gadolinium-DOTA nanoparticles in mice, signal enhancements in several organs including the lung were measured with ultrashort-echo-time-proton-MRI. The signal change over time in the different organs demonstrated the passage of the nanoparticles from the lung to the blood, then to the kidneys, and finally to the bladder [24].

### Toxicological aspects of nanomaterials

Toxic effects of nanoparticles are a major concern in pulmonary medicine. Especially ultrafine particles of low soluble, low toxic materials like titanium dioxide, carbon black, and polystyrene are overall more toxic and inflammatory than fine particles of the same material. This applies to both synthesized nanoparticles and natural dusts [25]. For nano related toxicity multiple mechanisms seem to be important. In the following, the interaction with the immune system, the creation of oxidative stress, and toxic effects on the genome are taken a closer look at. In order to correlate toxic effects with nanoparticle properties, it is necessary to thoroughly characterize the selected nanoparticles prior to administration.

#### Nanoparticle characterization

The most commonly used methods to characterize nanoparticles for toxicology studies are transmission electron microscopy (TEM) for size, morphology, and agglomeration, dynamic light scattering (DLS) for the size distribution of the particles, zeta potential measurement for nanoparticle surface charge, and x-ray diffraction (XRD) for the particles’ crystal structure. In some cases such as gold and silver nanoparticles, UV-vis spectroscopy can be used to determine size and size distribution due to a special size dependent optical activity [26]. Ideally, nanoparticle characterization is repeated after administration as changes of the nanoparticles during the application process are possible. In in-vitro experiments, nanoparticles are usually applied by mixing with cell culture medium. The dissolved components of the medium, especially the ions, lead to agglomeration and precipitation of many nanoparticles, causing significant changes in their physicochemical properties. Similar effects are to be expected when nanoparticles come into contact with surfactant or other biological fluids. It was shown, that some nanoparticles tend to form protein coronae in biological systems [27].

#### Effects on immune system and inflammation

**Fig. 5:**
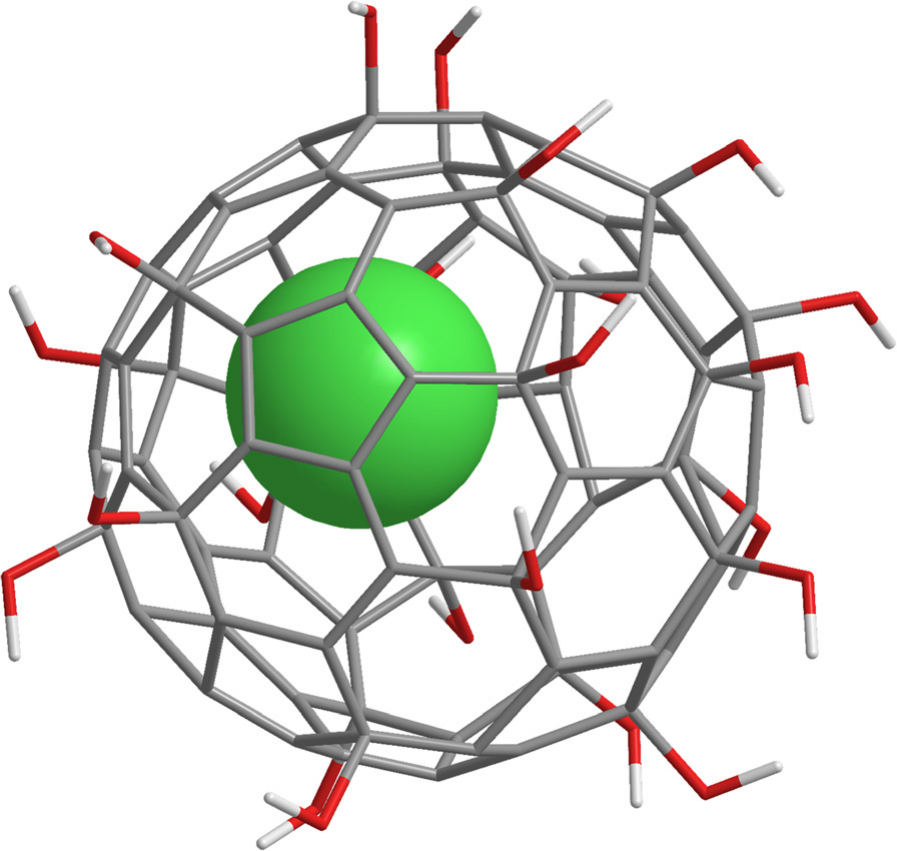
Gd@C_82_(OH)_22_ consists of a gadolinium atom (green) inside a 2 nm cage of carbon atoms (grey). The hydroxyl groups (red) outside the cage are responsible for water solubility. In water the molecule forms aggregates [Gd@C_82_(OH)_22_]_n_ with average size of 25 nm

Many nanoparticles possess properties that give them the potential to influence the immune system. In this context, nanoparticles’ ability to penetrate cellular boundaries, to escape phagocytation by macrophages, to act as haptens, and even to disturb the Th1/Th2 balance might be essential [28]. For carbon black nanoparticles, a recent study investigated the effects of inhalative exposure on mice with bleomycin-induced pulmonary fibrosis. The analysis of histology as well as cytokine expression suggested that the nanoparticles triggered an inhalation exacerbated lung inflammation. The author concluded that especially for people with pulmonary preconditions inhalation of nanoparticles can lead to serious health problems [29]. In this context, another study found out that PEGylated cationic shell-cross-linked knedel-like (cSCK) nanoparticles produced significantly less airway inflammation than non-PEGylated ones. This was explained by a change in endocytosis. In contrast to the clathrin-dependent endocytosis of non-PEGylated particles, the PEGylated cSCK nanoparticles showed a clathrin-independent route [30]. On the other hand, some nanomaterials exhibit impressive immune modulating activity. As an example, [Gd@C_82_(OH)_22_]_n_, a fullerene derivate with a gadolinium atom inside showed anticancer activity without being cytotoxic (Fig. 5). In vitro studies demonstrated that [Gd@C_82_(OH)_22_]_n_ activated dendritic cells (DCs) and even induced phenotypic maturation of those cells. Moreover, the [Gd@C_82_(OH)_22_]_n_ treated DCs also stimulated allogenic T cells in a Th1 characteristic. The effect of [Gd@C_82_(OH)_22_]_n_ was comparable, probably even stronger than the effect of lipopolysaccharide (LPS) on DCs. The study also verified that the nanoparticles were free of LPS contamination. In-vivo experiments on ovalbumin (OVA) immunized mice showed enhanced immune responses comparable to the adjuvant effect of Alum on OVA mice. However, whereas Alum lead to a Th2 response pattern with IL-4, IL-5 and IL-10 upregulation, [Gd@C_82_(OH)_22_]_n_ caused a Th1 pattern with upregulation of IFNγ [31]. Similar results were demonstrated in another study using a murine asthma model. OVA sensitized mice that were additionally treated with the nanomaterial graphene oxide during allergen sensitization had stronger airway remodeling and hyperresponsiveness than mice that have only been treated with OVA. The graphene oxide lead to a downregulation of Th2 dependent markers such as IL-4, IL-5, IL-13 IgE and IgG1 but increased Th1-associated IgG2a. Moreover, the graphene oxide increased the macrophage production of mammalian chinitases, chitinase-3-like protein 1 (CHI3L1), and AMCase, which could be the reason for the overall augmentation in airway remodeling and hyperresponsiveness [32]. However, this kind of immune modulation can also be utilized for therapeutic purposes. In a recent study a nanoparticle-based vaccine has been used to treat dust mite allergies in mice. The immune-modulating carriers were generated by loading dust mite allergen Der p2 and the potent Th1 adjuvant unmethylated cytosine-phosphate-guanine (CpG) into biodegradable poly(lactic-co-glycolic acid) (PLGA) polymer particles. Mice treated with those nanoparticles showed significantly lower airway hyperresponsiveness as well as lower IgE antibody levels after a 10 day intranasal Der p2 instillation compared to the control group. The authors conclude that this biodegradable nanoparticle-based vaccination strategy has significant potential for treating HDM allergies [33].

#### Oxidative stress and catalysis

Oxidative stress is often brought in context with nanotoxicology. It can be measured directly with dichlorofluorecein or indirectly by the upregulation of reactive oxygen species (ROS) eliminating enzymes like superoxide dismutase [34]. Another approach involves tests whether the nanoparticle dependent toxicity can be reduced by the application of an antioxidant. Widely used semiconductor materials such as lead sulfide nanoparticles may have the potential to generate oxidative stress in the lung. A recent study tested the toxicity of intratracheally applied 30 nm and 60 nm lead sulfide nanoparticles on rats. Oxidative damage was evaluated based on superoxide dismutase, total antioxidant capacity, and concentration of malondialdehyde. In addition to inflammatory responses, both 30 nm and 60 nm groups showed increased oxidative damage compared to control groups. The effect was significantly stronger for the 30 nm lead sulfide compared to the 60 nm nanoparticles [35]. Another nanomaterial which is associated with oxidative stress is nanosized titanium dioxide. Li et al. induced pulmonary injury in mice by daily intranasal instillation of suspended 294 nm TiO_2_ nanoparticles for 90 days, demonstrating that the rate of reactive oxygen species (ROS) generation increased with increasing TiO_2_ doses. Moreover lipid, protein and DNA peroxidation products were identified in elevated doses, which suggests that ROS dependent lung damage was significant in the nanoparticle treated animals [36]. Furthermore, in vitro tests on BEAS-2B and A549 lung cell lines demonstrated that the commonly used nanoparticles ZnO and Fe_2_O_3_ are very different in terms of creating oxidative stress. The Fe_2_O_3_ nanoparticles with an average diameter of 39 nm were distributed in the cytoplasm, whereas the 63 nm ZnO nanoparticles were trapped in organelles such as the endosome. In contrast to the Fe_2_O_3_ nanoparticles the ZnO nanoparticles caused reactive oxygen species production as well as cell cycle arrest, cell apoptosis, mitochondrial dysfunction and glucose metabolism perturbation [37] (Table 1).

**Table 1:** Oxidative stress induction in respiratory tissue by different nanoparticles

Nanoparticles	PbS	PbS	TiO2	ZnO	Fe_2_O_3_
Size	60 nm	30 nm	294 nm	63 nm	39 nm
Model	in vivo	in vivo	in vivo	in vitro	in vitro
	(rat lungs)	(rat lungs)	(mice lungs)	(BEAS-2B and A549 lung cells)	(BEAS-2B and A549 lung cells)
Oxidative Stress	+	++	+	+	-

#### Genotoxicity

Another important type of toxicity caused by nanoparticles is genotoxicity. A common method to quantify genotoxicity is the comet assay, which uses electrophoresis to detect DNA strand breaks. This assay was used in a recent study to check whether intratracheal instilled fullerene C_60_ nanoparticles induced DNA damage in male rats. However, despite inflammatory responses and hemorrhages in the alveoli of the C_60_ treated rats, there was no significant increase in fractured DNA in their lung cells. Therefore, it was concluded that even at inflammation inducing doses, fullerene C_60_ nanoparticles have no potential for DNA damage in the lung cells of rats [38]. Similarly, another study demonstrated that intratracheal instillation of anatase TiO_2_ nanoparticles on rats did not result in genotoxicity. None of the TiO_2_ groups showed an increase in fractured DNA while the positive control with ethyl methanesulfonate exhibited significant increases [39]. In contrast to those results, Kyjovska ZO et al. found that even in low doses, where no inflammation occurs, Printex 90 carbon black nanoparticles induce genotoxicity in mice. There was no inflammation, cell damage and acute phase response, which means that the increased DNA strand breaks are related to direct DNA damage caused by the nanoparticles [40]. On the other hand, a recent study suggests that CeO_2_ nanoparticles may be even used as antioxidant and anti-genotoxic agents in the lung. After treatment with the oxidative stress-inducing agent KBrO_3_, BEAS-2B cells pretreated with the CeO_2_ nanoparticles showed significantly less intracellular ROS as well as a reduction in DNA damage compared to non-pretreated cells [41].

### Biodistribution

#### Nanoparticle detection

Research on the biodistribution of nanoparticles requires tracking of the applied nanoparticles in the test animal. Conventional light microscopy is not able to detect nanoparticles because of Abbe’s law. Therefore, electron microscopic imaging is often required. However, light microscopy can be used to describe the nanoparticle induced changes in the cell morphology without being able to see the nanoparticles themselves. Additionally, nanoparticles can be indirectly made detectable in light microscopy by a method called autometallography. This is a silver staining that can be used to increase the size of several types of nanoparticles like gold, silver, and some metal sulfides and selenides in the histological section [42]. This technique was used to detect silver nanoparticles in the olfactory bulb and lateral brain ventricles of mice that had been intranasally treated with 25 nm silver nanoparticles [43].

#### Particle deposition and resorption in the respiratory tract

**Fig. 6:**
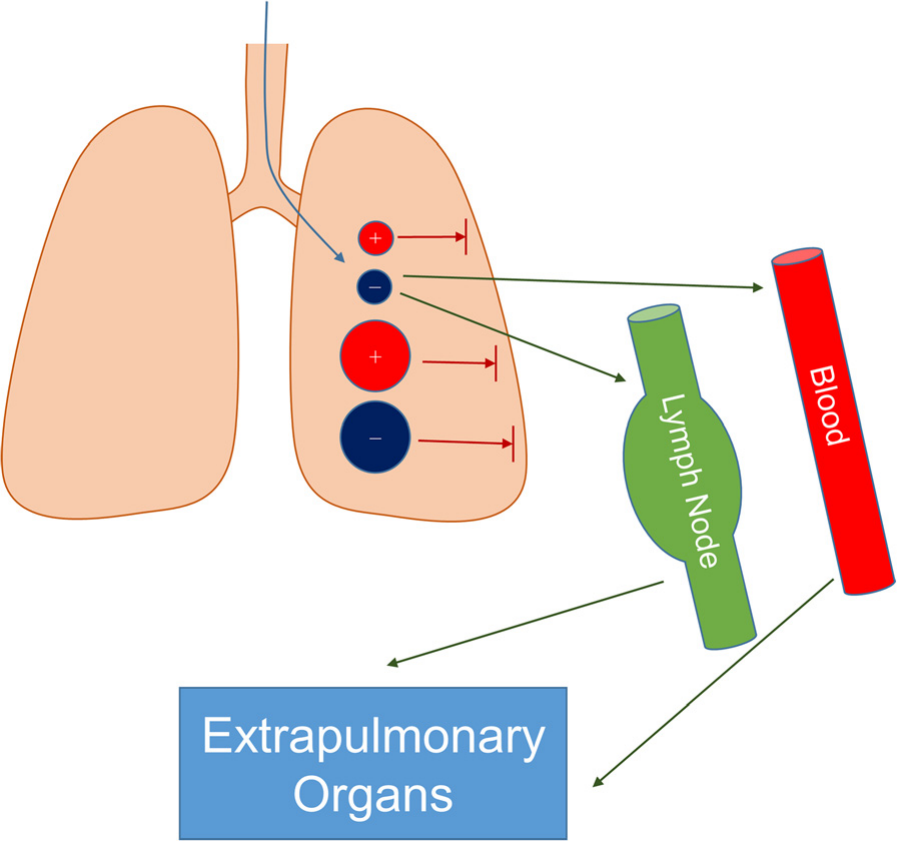
Pulmonary uptake of nanoparticles depends on size and surface charge. Positively charged nanoparticles and nanoparticles that are bigger than 34 nm cannot pass the epithelial barrier of the lung. Only small and not positively charged nanoparticles can translocate from the lung over blood and lymph system to the organism

Most research about biodistribution of nanoparticles in organisms focuses on intravenous injection. However, nanoparticles were shown to be able to pass the blood air barrier of the lung. Whether or not nanoparticles can travel through the lung into the body seems to be size dependent. This was evaluated by injecting neutron activated radioactive gold nanoparticles of 1.4 nm and 18 nm intratracheally to rats. The bigger nanoparticles almost completely retained in the lung while significant amounts of the smaller 1.4 nm particles were found in blood, liver, skin and carcass 24 h after instillation [44]. Choi H. S. et al. applied nanoparticles of different size and charge to mice. The nanoparticles were tracked in different organs through fluorescence labeling. It was demonstrated that nanoparticles rapidly translocated to the mediastinal lymph nodes if they possess a hydrodynamic diameter of 34 nm or less and a neutral or anionic surface. Bigger and positively charged nanoparticles exhibited no significant uptake [45] (Fig. 6). In addition to physical parameters of the applied nanoparticles the health status of the exposed organism also seems to play an important role. A recent study showed that the distribution of oropharyngeal instilled 40 nm gold nanoparticles is influenced by additional LPS treatment. The gold content of organs was measured with inductively coupled plasma mass spectroscopy. BALB/C mice that had been oropharyngeal treated with LPS 24 h prior to the nanoparticle administration exhibited less gold content in their lungs than untreated mice. In both groups gold was detected in different organs. High concentrations were found in heart and thymus in the non LPS group, while the LPS treated mice accumulated most of the gold in the spleen. The author concluded that nanoparticle uptake may depend on medical preconditions [46].

## Conclusions

Over the last decade, major breakthroughs in nanotechnology have been achieved. It is only a matter of time before new nano based drugs reach respiratory medicine. Especially the fields of targeted drug delivery, gene therapy, and hyperthermia offer great potential for modern drugs. On the other hand the increased use of nanomaterials in all fields of life also bears the risk of exposure through inhalation. It is therefore essential to understand pulmonary toxicology of nanomaterials in all its facets. However, it is still very unclear why the toxic effects of nanoparticles in the respiratory tract are so inhomogeneous and not well predictable. In this context, not only local reactions of lung and airways but also nanoparticle uptake and distribution in the organism are important factors and therefore fields of current research. As only few nanoparticle compositions have been tested, it is questionable whether those results can be easily adapted to other nanoparticles. Because of the continuously increasing diversity of engineered nanoparticles, toxicology can hardly keep pace with the safety assessment of future products. Therefore, more attention should be set on this wide field of research.

## Abbreviations

CHI3L1: Chitinase-3-like protein 1
cSCK: Cationic shell-cross-linked knedel-like
CT: Computer tompgraphy
DC: Dendritic cell
DLS: Dynamic light scattering
EGFR: Epidermal growth factor receptor
EPR: Enhanced permeability and retention
LPS: Lipopolysaccharide
MRI: Magnetic resonance imaging
NSCLC: Non-small-cell lung carcinoma
OVA: Ovalbumin
PEG: Polyethylene glycol
PLA: Polylactic acid
PLGA: Poly(lactic-co-glycolic acid)
PSMA: Prostate-specific membrane antigen
ROS: Reactive oxygen species
TEM: Transmission electron microscopy
TNF-α: Tumor necrosis factor alpha
XRD: X-ray diffraction
